# Transcriptomic analysis of isolated and pooled human postmortem cerebellar Purkinje cells in autism spectrum disorders

**DOI:** 10.3389/fgene.2022.944837

**Published:** 2022-11-09

**Authors:** Cheryl Brandenburg, Anthony J. Griswold, Derek J. Van Booven, Michaela B. C. Kilander, Jeannine A. Frei, Michael W. Nestor, Derek M. Dykxhoorn, Margaret A. Pericak-Vance, Gene J. Blatt

**Affiliations:** ^1^ Hussman Institute for Autism, Baltimore, MD, United States; ^2^ University of Maryland School of Medicine, Baltimore, MD, United States; ^3^ John P. Hussman Institute for Human Genomics, University of Miami, Miami, FL, United States

**Keywords:** autism, cerebellum, purkinje cells, extracellular matrix, nervous system development, cell migration, axon guidance, immune function

## Abstract

At present, the neuronal mechanisms underlying the diagnosis of autism spectrum disorder (ASD) have not been established. However, studies from human postmortem ASD brains have consistently revealed disruptions in cerebellar circuitry, specifically reductions in Purkinje cell (PC) number and size. Alterations in cerebellar circuitry would have important implications for information processing within the cerebellum and affect a wide range of human motor and non-motor behaviors. Laser capture microdissection was performed to obtain pure PC populations from a cohort of postmortem control and ASD cases and transcriptional profiles were compared. The 427 differentially expressed genes were enriched for gene ontology biological processes related to developmental organization/connectivity, extracellular matrix organization, calcium ion response, immune function and PC signaling alterations. Given the complexity of PCs and their far-ranging roles in response to sensory stimuli and motor function regulation, understanding transcriptional differences in this subset of cerebellar cells in ASD may inform on convergent pathways that impact neuronal function.

## Introduction

A diagnosis of autism spectrum disorder (ASD) is accompanied by both gifts and challenges and the description of ASD as a “spectrum” acknowledges the heterogeneous presentation of symptoms across individuals. The American Psychiatric Association’s Diagnostic and Statistical Manual (DSM-5) provides standardized criteria that outline typical differences in behaviors that fall into the broad categories of social communication and interaction and restricted and repetitive behaviors (American Psychiatric Association, 2013). Within the category of restricted and repetitive behavior, sensorimotor challenges play a prominent role in diagnosis. For example, many individuals display stereotyped and repetitive motor movements that include hand flapping, rocking, pacing, spinning objects, lining up toys, echolalia, etc. that is often accompanied by hyper- or hyporeactivity to sensory input. Although attention is usually focused on the differences in complex behaviors of social communication, there is a large body of research that implicates alterations in fundamental motor patterns in ASD. Basic motor differences during development may translate into challenges with complex behaviors that rely on the acquisition of fundamental motor skills (for review, see: Subramanian et al., 2017). The underlying circuitry within the brain that is responsible for mediating such a diverse set of motor and cognitive behaviors has not been fully defined, however, there is growing consensus that the cerebellum plays a major role in ASD (Fatemi et al., 2012; Becker and Stoodley, 2013; Rogers et al., 2013; Wang et al., 2014; Hampson and Blatt, 2015). As knowledge of the cerebellum expands to include functional roles in non-motor domains as well as the typical motor-domains (Schmahmann, 2019), it is now clear that the cerebellum influences a wide range of behaviors relevant to the ASD phenotype.

Aside from the many cerebellar disorders that have comorbid ASD associated symptoms (Becker and Stoodley, 2013; Wang et al., 2014), alteration of cerebellar circuitry early in development is correlated with ASD (Hashimoto et al., 1995; Courchesne et al., 2001; Beversdorf et al., 2005; Limperopoulos et al., 2007). Cerebellar insult (prenatal stressors, infection, etc.) during development has an ASD risk ratio comparable to the risk seen with twin studies and the strongest genetic associations (i.e. highest-risk single genetic variants) (Wang et al., 2014). Furthermore, imaging studies repeatedly implicate the cerebellum in structural differences in ASD, where volumetric differences emerge in the early years of life, persist into adulthood and have been associated with language deficits (Scott et al., 2009; Becker and Stoodley, 2013; Wang et al., 2014; Hampson and Blatt, 2015).

Importantly, when unbiasedly assessing the resting-state functional connectome in ASD across the entire brain, the cerebellum emerged as the only region meeting stringent criteria for significant abnormal connectivity (Arnold Anteraper et al., 2019). These converging lines of evidence from behavioral, lesion and imaging studies implicate the cerebellum in the development of ASD and highlight the need for a deeper understanding of how the cerebellum mediates sensorimotor and cognitive behavior. This will likely be a crucial step toward developing support for ASD individuals and points to the need for understanding the specific cerebellar circuitry deficits that lead to the various reported differences.

Purkinje cells (PCs) are central to cerebellar function as the only output cells of the cerebellar cortex. Human postmortem studies have shown differences in PC number (Bauman and Kemper 1985; Bauman et al., 1995; Bailey et al., 1998; Whitney et al., 2008; Skefos et al., 2014), size (Fatemi et al., 2002) and gene expression (Yip et al., 2007; Yip et al., 2008; Soghomonian et al., 2017) in the ASD brain. PC dysfunction is the most consistent neuropathological finding to date, with as many as 75% of cases showing reductions in number (Schumann and Nordahl, 2011; Hampson and Blatt, 2015).

In ASD, many of the genes implicated by genome-wide association studies (GWAS) affect synaptic stability and adhesion molecules (Hussman et al., 2011; Lin et al., 2016). Since PCs have hundreds of thousands of synapses, making their dendritic branching the most complex in the brain, mutations in synaptic proteins and adhesion molecules may be particularly detrimental to PC signaling and survival. The abundant spines on PCs modify their shape and function in response to stimuli and have been shown to be involved in cerebellar motor learning (Kleim et al., 1998; Kim et al., 2002; Federmeier et al., 2002; Lee et al., 2005). Therefore, it’s possible their disruption can provide a mechanism behind the decreased numbers of PCs and alterations reported in human postmortem ASD studies.

An important study (Parikshak et al., 2016) compared transcriptome alterations between brain regions in ASD, concluding that the cortex is more vulnerable to transcript alterations than the cerebellum. However, it is possible that the heavily skewed proportion toward granule cell number as compared to PC number represented in bulk cerebellar tissue obscures differences in PC transcriptome profiles, as it is surprising there would be few transcriptional differences in a brain region as heavily implicated in ASD as the cerebellum. Until recently, most studies investigating altered gene expression in human postmortem brain tissue have been limited to heterogeneous cell populations acquired from whole tissues. However, laser capture microdissection has emerged as a viable method to extract single cell types from the brain (Murray, 2007; Chu et al., 2009; Pietersen et al., 2009; Cunnea et al., 2010; Greene et al., 2010; Pietersen et al., 2011; Simpson et al., 2011; Waller et al., 2012).

Given the numerous studies implicating PC pathophysiology in ASD, we used laser capture microdissection to isolate and extract PCs from the billions of other cells in the cerebellum and compared the transcriptional profile of PCs in ASD cases to controls. We identified 427 differentially expressed genes, which were enriched for gene ontology (GO) biological processes related to nervous system development, synaptic and cellular signaling, immune responses, extracellular matrix organization and MAPK signaling. The number of DEGs is consistent with those found in cortical studies (Parikshak et al., 2016), which highlights the importance of single-cell analysis in complex disorders. Similarly, single nucleus RNA sequencing of postmortem ASD brains in cortical regions resulted in 513 differentially expressed genes, in which transcripts of neurons were more impacted than that of glial cells (Velmeshev et al., 2019), however, metanalysis of single cell RNA sequencing data suggests glia may also be impacted in ASD (Nassir et al., 2021). The data presented provides evidence for disrupted neuronal function in mature cells while also suggesting that developmental processes and early connectivity mechanisms are impacted.

## Methods

### Postmortem tissue

Human postmortem brain tissue was obtained from the University of Maryland Brain and Tissue Bank, a brain and tissue repository of the NIH Neurobiobank. Case demographics are provided in Table 1. All ASD cases had confirmed diagnoses through Autism Diagnostic Interview-Revised (ADI-R) scores and/or received a clinical diagnosis of autism from a licensed psychiatrist. Cases were age- and PMI-matched, but different race/ethnic backgrounds were taken as available. As this research did not involve live human subjects, Institutional Review Board approval and informed consent were not necessary. However, the University of Maryland Brain and Tissue Bank (NIH Neurobiobank) is overseen by Institutional Review Board protocol number HM-HP-00042077 and de-identifies all cases before distribution to researchers.

**TABLE 1:** Postmortem case demographics.

Case #	Diagnosis	Age at Death (Y)	Cause of Death	Self-Reported Ancestry	Sex	PMI (H)
914	Control	20	Vehicle accident, multiple injuries	Non-Hispanic White	M	18
1158	Control	16	Cardiomegaly	Non-Hispanic White	M	15
4599	Control	23	Cardiac arrthymia/anomalous coronary artery	African American	M	18
5079	Control	33	Drowning complicated by alcohol intoxiaction	Non-Hispanic White	M	16
5113	Control	36	Pulmonary embolism	African American	M	20
5163	Control	14	Drowning	Non-Hispanic White	M	12
5170	Control	13	Gun shot of chest	African American	M	20
5334	Control	12	Hanging/Suicide	Hispanic	M	15
5387	Control	12	Drowning	Non-Hispanic White	M	13
5391	Control	8	Drowning	Non-Hispanic White	M	12
5566	Control	15	Hypertrophic cardiomyopathy	African American	F	23
5646	Control	20	Reactive ariway disease	Non-Hispanic White	F	23
5669	Control	24	Hypertensive cardiovascular disease	African American	F	29
5705	Control	31	Cardiac Arrhythmia	Non-Hispanic White	M	26
5813	Control	20	Atherosclerotic Cardiovascular disease	African American	M	24
5889	Control	27	Acute Pneumonia complicated by sepsis	Non-Hispanic White	M	12
5893	Control	19	Dilated Cardiomegaly	Non-Hispanic White	M	11
5922	Control	46	Atherosclerotic Cardiovascular disease	Non-Hispanic White	M	10
5926	Control	21	Cardiac Arrhthymia w/probable sickle cell disease	African American	M	27
	Average age 21.58			Average PMI 18.11		
	stdevp 9.32			stdevp 5.75		
	Mann Whitney *U* test 0.28			Mann Whitney *U* test 0.08		
4334	ASD	11	Acute Hemorrhagic Tracheobronchitis	Hispanic	M	27
4849	ASD; Language Delay; Lead Poisoning	7	Drowning	African American	M	20
4999	ASD; Intellectual Disability Severe	20	Cardiac Arrhythmia	Non-Hispanic White	M	14
5027	ASD	37	Obstruction of bowel due to adhesion	African American	M	26
5115	ASD; Epilepsy Temporal	46	Complications of Pseuodmyxoma Peritonei	Non-Hispanic White	M	29
5144	ASD	7	Cancer	Non-Hispanic White	M	3
5176	ASD; Subdural Hematoma	22	Subdural hemorrhage	African American	M	18
5278	ASD; Epilepsy	15	Drowning associated with seizure disorder	Non-Hispanic White	F	13
5294	ASD	19	Suicide, hanging	Non-Hispanic White	M	16
5297	ASD; Intellectual Disability NOS	33	Asphyxia due to face down position	Non-Hispanic White	M	50
5308	ASD	4	Skull Fractures	Non-Hispanic White	M	21
5403	ASD	16	Caridac Arrhythmia	Non-Hispanic White	M	35
5419	ASD	19	Natural/epilepsy	Non-Hispanic White	F	22
5565	ASD; Epilepsy	12	Seizure Disorder, Complications	African American	M	22
5841	ASD; Attention-Deficit/Hyperactivity Disorder NOS	12	Hanging	Non-Hispanic White	M	15
5878	ASD	27	Peritonitis	Non-Hispanic White	M	42
5940	ASD; Epilepsy	29	Epilepsy complicated by drowning	Non-Hispanic White	M	20
5945	ASD; Epilepsy; Developmental Delay	20	CPA	Non-Hispanic White	M	24
5978	ASD; Attention-Deficit/Hyperactivity Disorder NOS	11	Smoke inhalation	Non-Hispanic White	M	21
6033	ASD; Epilepsy	14	Seizure Disorder	Non-Hispanic White	F	25
	Average age 19.05			Average PMI 23.15		
	stdevp 10.52			stdevp 10.12		

Overall, 39 samples were used for this analysis. This number was primarily dictated by the paucity of autopsy samples available from younger individuals and secondarily by the amount of time taken for the extraction of single cells from each sample by laser capture microdissection. Twenty ASD and nineteen control fresh frozen age, gender and postmortem interval (PMI) matched human lateral hemisphere (crus II) cerebellar blocks were dissected in a consistent anatomical location across cases and cut at 8 µm onto membrane slides (Zeiss MembraneSlide 1.0 PEN 41590-9041-000) with a cryostat (Leica CM 1950) and kept frozen at −80°C.

### Laser capture microdissection

Sections (8 µm) on membrane slides were briefly fixed in 75% ethanol, dipped into RNase-free water, exposed to a rapid Cresyl violet solution and placed in a series of alcohols for dehydration as described (41). A Zeiss PALM MicroBeam laser microdissection system was used to manually outline PCs, as identified by morphology and cerebellar architecture, which were then lifted into an adhesive cap, effectively isolating approximately 1,500 PCs per case from the surrounding cerebellar tissue. The cells were lysed, and RNA extraction was performed with a Qiagen RNeasy Micro Kit (74004).

### RNA sequencing

RNA sequencing was performed at the John P. Hussman Institute for Human Genomics Center for Genome Technology at the University of Miami. RNA integrity (RIN) scores and RNA concentration were determined for the extracted RNA using the 2100 BioAnalyzer (Agilent Technologies, Santa Clara, CA) and the Qubit RNA High Sensitivity Assay Kit (Thermo Fisher Scientific, Waltham, MA). Samples with a RIN ≥4 were included for library preparation and sequencing. Total RNA was prepared using the Ovation SoLo RNA-Seq Library Preparation Kit (Tecan Life Science, Mannedorf, Switzerland). Sequencing was performed on the Illumina NovaSeq 6,000 (Illumina, San Diego, CA) with single end 100bp reads targeting 25 million reads per sample.

### Primary RNAseq bioinformatics

Raw FASTQs were processed through a bioinformatics pipeline including adapter trimming by TrimGalore (v0.6.1) (https://github.com/FelixKrueger/TrimGalore), alignment with the STAR aligner 2.5.0a{{43692 Dobin, A. 2013}} to the GRCh38 human reference genome, and gene counts quantified against the GENCODE v35 human gene release using the GeneCounts function in STAR. Alignment quality control was performed with the CollectRnaSeqMetrics module implemented in Picard v1.103 (http://broadinstitute.github.io/picard). Protein coding genes that had a raw read count <10 in 25% or more of samples were filtered out from further analysis.

### Differential gene expression

The gene counts for each sample were transformed and normalized using the variance-stabilizing transformation method implemented in the Biocinductor package DESeq2 (v2.2){{47440 Love,M.I. 2014}} run with R software v3.4.3. Principle linear regression models within DESeq2 were used to test differentially expressed genes between individuals with ASD and controls, correcting for covariates of age, self-reported ethnicity, sex and post-mortem interval. We analyzed the differentially expressed genes for enriched pathways using the GO Biological Processes and KEGG pathway databases using the DAVID analysis tool (Dennis et al., 2003) with the background set to only those genes analyzed in this experiment. To identify potential protein interaction networks between differentially expressed genes, we utilized the STRINGdb database (Snel et al., 2000; Szklarczyk et al., 2019) accessed at https://string-db.org/with parameters of high confidence interaction (0.7) and MCL using a stringent inflation parameter of 1.5. Bioinformatic analysis on the defined clusters was performed to identify GO processes that are overrepresented in the genes that make up each cluster.

## Results

### RNAseq quality control

**FIGURE 1:**
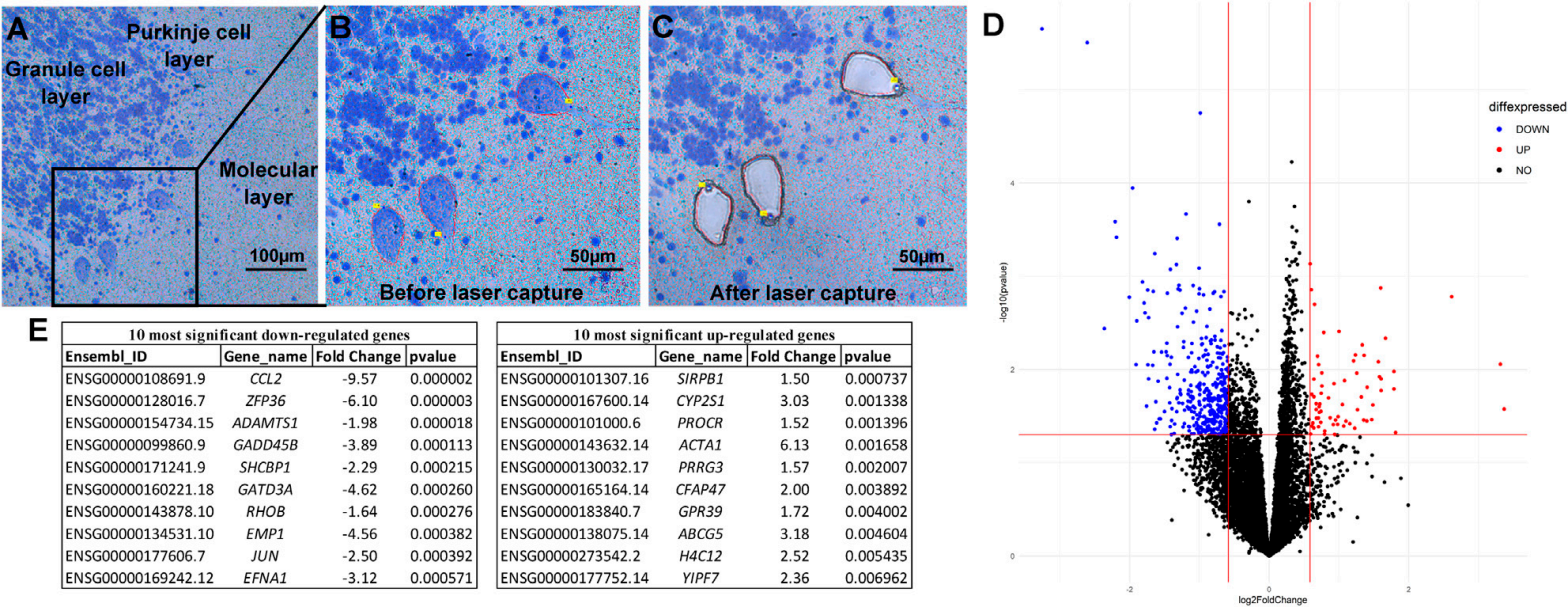
Laser capture microdissection and differential expression analysis. Tissue is stained with a rapid Nissl protocol, which clearly delineates Purkinje cells based on the consistent architecture of the cerebellar cortex and large cell bodies **(A)**. The Zeiss RoboPalm software is used to manually outline individual thinly sliced (8 µm) Purkinje cells **(B)** and specifically removes the cell body, leaving the other cells and tissue behind **(C)**. After RNA extraction and sequencing, Principle Linear regression models within DESeq2 were used to test differentially expressed genes between individuals with ASD and controls, correcting for covariates of age, self-reported ethnicity, sex, and post-mortem interval **(D)**. The top ten significant up- and down-regulated genes are provided **(E)**.

Each 8 µm section was stained with a rapid Cresyl violet stain to visualize cell morphology and outline PC bodies (Figures 1A,B). Upon removal from the tissue (Figure 1C), 1,500 PCs, on average, were pooled and the RNA was immediately extracted. RNAseq data was of high quality and there were no significant difference between the ASD and the control samples in QC metrics, including number of total reads, percentage of uniquely mapping reads, and percentage of reads mapping to the transcriptome (Supplementary Material S1, Table 1). Each sample generated on average 34.6 ± 6.9 million reads with no significant difference in the number of reads between groups (Student’s *t*-test, *p* = 0.15). An average of 61.0 ± 9.8% of total reads mapped uniquely to the reference genome and of those 65.1 ± 5.5% mapped to annotated genes, again with no significant differences between ASD and control samples (Student’s *t*-test, *p* = 0.58 and *p* = 0.33, respectively). Of the 19,954 protein coding genes in the GENCODE v35, 13,702 were detected with at least ten reads in at least 25% of the samples. Principal component analysis (PCA) of the variance stabilized transformed values indicated a clear separation of sex based on gene expression, but no outlier samples beyond 2.5 standard deviations from the mean nor distinction of samples by status or self-reported ethnicity (Supplementary Figure S1). Since PCs are the only cell type within the PC layer and the sections were thin, it is unlikely that other cell types were included in extracted cells. To confirm this, we compared the average normalized expression of genes associated with cerebellar PCs versus those associated with cerebellar astrocytes or oligodendrocytes (Kuhn et al., 2012) (Supplementary Figure S2). The average expression of the PC associated genes was significantly higher than those associated with astrocytes or oligodendrocytes (Kruskal-Wallis *p*-value < 0.0001).

### Differential expression analysis

**FIGURE 2:**
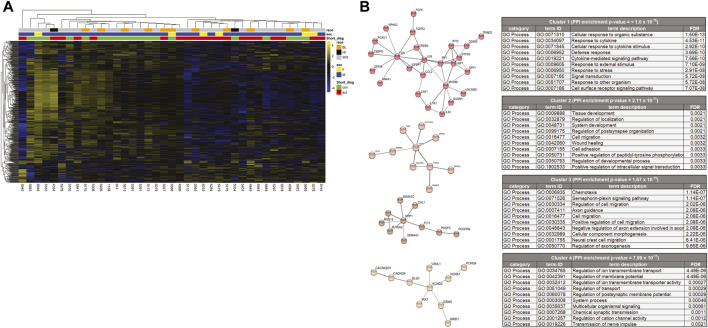
**(A)** Heat map of differentially expressed genes. Of the total 427 differently expressed genes, 360 were down-regulated and 67 were up-regulated in ASD cases. Both the genes (rows) and cases (columns) were clustered using dendrograms constructed using Euclidean distances and hierarchical clustering and grouped by case demographics. **(B)** STRINGdb analysis of the differentially expressed genes that were decreased in the cases compared to the controls. The network was divided into clusters using the MCL inflation parameter and the top 4 clusters were analyzed for enrichment of Gene Ontology processes and the top 10 processes for each cluster are represented.

Using the criteria of nominal *p*-value ≤ 0.05 and a fold change of >1.5, a total of 427 protein coding genes were found to be differentially-expressed between the ASD and control samples (Figure 1D). The 10 most significantly differentially up and down-regulated genes, as determined by *p*-value are listed in Figure 1E (and Supplementary Figure S3). Of these 427 differentially expressed genes, 360 were down-regulated and 67 were up-regulated in ASD compared to controls (Figure 2A). A complete list of all genes tested and all genes that are significantly differentially expressed is provided in Supplementary Tables S2,3, respectively. These include differential expressed genes (followed by their ASD gene score)—*ADCY3(2), AGAP2(2), APBA2(2), BCAS1(2), CACNA2D1(2), CADPS2(2), CDH10(2), CDKL5(1), DLG1(2), GLRA2(2), GRID1(2), GRM5(2), KCND2(2), LRRC4(2), NAV2(2), NR4A2(1), NTNG1(2S), P2RX5(2), PCDH19(1), PCDHA2(2), PRUNE2(2), RANBP17(2), RELN(1),* and *RIMS1(1)*—that are among the autism candidate genes list in the human gene module of SFARI Gene (https://gene.sfari.org/) with a score of 1 (High Evidence) or 2 (Strong Candidate), with S in each category indicating genes classified as syndromic. SFARI curates lists of all genes implicated in ASD from GWAS and other genetic studies.

### Pathway analysis

To better understand how these differentially expressed genes impact PC function, GO term and KEGG pathway enrichment analyses were performed (Table 2 and Supplementary Figure S4). The differentially expressed genes were enriched for GO biological processes broadly falling into categories of neuron development, signal transduction, extracellular matrix organization and synaptic function. The KEGG pathways were enriched for MAPK signaling (Table 2). In order to provide more functional context to these altered genes, we performed protein-protein interaction analyses using STRINGdb. STRINGdb analysis was performed on the differentially expressed genes of cases compared to controls as two separate analyses for increased or decreased genes. 352 of the 427 differentially expressed genes were down-regulated and produced a protein-protein interaction cluster with a PPI enrichment *p*-value of 1.1 × 10^-14^ (Supplementary Figure S5). This parent cluster was enriched for 216 GO processes (false discovery rate (FDR) < 0.05 (Supplementary Material S1). These included processes related to nervous system development (neurogenesis), cell communication, cell migration, cell adhesion, and axon development. This parental PPI cluster could be broken down into 26 subclusters that had a PPI enrichment *p*-value < 0.005. The clusters which involved >10 proteins are shown in Figure 2B along with the top 10 GO processes enriched in each cluster. Cluster one was enriched for 137 processes which included multiple processes related to immune responses (such as cytokine-mediated signaling pathways, innate immune response, and type I interferon pathway), cell metabolism and response to growth factors. Cluster two showed an enrichment for 43 processes, including cell migration, tissue/organ development, neurogenesis, cell-substrate adhesion and regulation of dendrite development. Cluster three was enriched in 68 GO processes which involved functions such as chemotaxis, Semaphorin-plexin signaling pathway involved in axon guidance, cell migration, signal transduction and cell development. Cluster four was enriched for 26 GO processes, including regulation of ion transmembrane transport, regulation of membrane potential, chemical synaptic transmission, and action potential. Other clusters were enriched for GO processes, such as apoptosis (Cluster 5), cAMP metabolism (Cluster 6), phospholipase C-activating G protein-coupled receptor signaling pathway (Cluster 7), GABA-A receptor activity (Cluster 8), neuron fate specification (Cluster 11) and negative regulation of sodium ion transmembrane transporter activity (Cluster 15). All of the GO processes enriched for each of the 26 clusters is shown in Supplementary Material S1.75 of the 427 differentially expressed genes were up-regulated in the cases compared to the control samples. However, STRINGdb analysis of the up-regulated genes showed no enrichment for GO processes (data not shown).

**TABLE 2:** Enriched pathways.

GO Biological Process (Top 25)					
Term	Count	%	*p*-value	Fold Enrichment	Genes
GO:0007411∼axon guidance	18	4.196	9.12E-06	3.6300	SLC7A11, SEMA3B, LAMA5, SEMA3C, NGFR, EFNA1, LGR6, KLF7, CHL1, NRP1, ETV1, NIBAN2, RELN, FLRT3, PAX6, EDN3, SEMA4C, LAMA3
GO:0007267∼cell-cell signaling	13	3.030	6.79E-05	4.1271	PHEX, FGFR3, SEMA3B, PCSK5, EFNA1, AGT, NRP1, FGF5, PANX2, CXCL14, GJB6, TNFSF10, EDN3
GO:0016477∼cell migration	19	4.429	1.05E-04	2.8873	LIMA1, SIRPA, CD44, TIAM1, BTG1, TYRO3, LAMA5, PDGFRB, HES1, EFNA1, TGFBR3, RHOB, FMNL3, FLT1, RHOV, PRAG1, LAMA3, NTNG1, IQGAP1
GO:1990138∼neuron projection extension	6	1.399	2.30E-04	10.1257	TIAM1, FLRT3, NRN1, PAK6, IQGAP1
GO:0014068∼positive regulation of phosphatidylinositol 3-kinase signaling	9	2.098	3.13E-04	5.1532	RELN, AGAP2, ERBB3, FLT1, PDGFRB, NEDD4, PDGFC, AGT, DCN
GO:0007218∼neuropeptide signaling pathway	8	1.865	3.27E-04	5.9655	NTSR2, GLRA2, SORCS1, TYRO3, GPR83, LTB4R, SSTR2, NXPH3
GO:0007268∼chemical synaptic transmission	16	3.730	4.45E-04	2.8822	SV2B, DLG1, KCND2, UNC13C, KCNA1, RPS6KA1, DOC2A, CHRNB4, HAP1, GLRA2, CBLN1, APBA2, GABRD, TPGS1, GRM5, GABRA6
GO:0009887∼animal organ morphogenesis	11	2.564	6.12E-04	3.7926	LAMA5, PAX6, MEIS3, PDGFC, CCL2, NRP1, FGF5, DCN, LFNG, LAMA3, NTNG1
GO:0042391∼regulation of membrane potential	10	2.331	7.47E-04	4.0588	CHRNB4, NTSR2, GLRA2, DLG1, GABRD, RIMS1, NEDD4, KCNA1, GABRA6, NEDD4L
GO:0006955∼immune response	13	3.030	9.58E-04	3.1107	IKBKE, SEMA3C, LTB4R, TGFBR3, TRIM22, HLA-DRA, IL1R1, TNFSF4, IFITM3, CXCL14, TNFSF10, CCL2, RAG1
GO:0071277∼cellular response to calcium ion	9	2.098	0.0014	4.1226	RYR3, CPNE2, JUN, FOS, AKR1C3, NEUROD2, SYT9, IQGAP1, CLIC4
GO:0001501∼skeletal system development	10	2.331	0.0015	3.6856	PHEX, GDF10, FGFR3, COL3A1, ACD, PRELP, COL9A2, COL10A1, FRZB, COL19A1
GO:0061098∼positive regulation of protein tyrosine kinase activity	6	1.399	0.0018	6.6341	AFAP1L2, RELN, ERBB3, EFNA1, GRM5, AGT
GO:0034765∼regulation of ion transmembrane transport	10	2.331	0.0019	3.5627	KCNQ4, HVCN1, KCND2, CACNA2D1, NEDD4, KCNK5, KCNJ6, KCNA1, CLIC4, NEDD4L
GO:0060078∼regulation of postsynaptic membrane potential	6	1.399	0.0021	6.4129	KCND2, GABRD, GRID1, GRM5, KCNA1, GABRA6
GO:0048146∼positive regulation of fibroblast proliferation	7	1.632	0.0028	4.8794	NGFR, PDGFRB, DDR2, PDGFC, JUN, AGT, S100A6
GO:0006937∼regulation of muscle contraction	4	0.932	0.0030	12.8258	TNNC1, MYL9, SSTR2, KCNA1
GO:0048839∼inner ear development	6	1.399	0.0038	5.6585	CXCL14, GJB6, BMPER, PDGFRB, NEUROD1, CEBPD
GO:0070374∼positive regulation of ERK1 and ERK2 cascade	11	2.564	0.0040	2.9640	NECAB2, FGFR3, CD44, BMPER, GLIPR2, PDGFRB, SPRY2, PDGFC, CCL2, JUN, NRP1
GO:0030198∼extracellular matrix organization	10	2.331	0.0042	3.1747	COL8A1, COL3A1, COL9A2, THSD4, COL10A1, FBLN2, B4GALT1, ADAMTS1, ADAMTS18, COL19A1
GO:0001819∼positive regulation of cytokine production	6	1.399	0.0055	5.1997	TNFSF4, CRTAM, IL33, C1QTNF3, AGT, PELI1
GO:0006954∼inflammatory response	14	3.263	0.0065	2.3627	CD44, LTB4R, FOS, SPP1, IL1R1, AFAP1L2, TNFSF4, MYD88, CSF1, NFATC4, NFKBIZ, CCL2, TNFRSF1A, IGFBP4
GO:0035924∼cellular response to vascular endothelial growth factor stimulus	5	1.166	0.0069	6.4129	FLT1, SPRY2, NR4A1, NRP1, GAS1
GO:0007166∼cell surface receptor signaling pathway	10	2.331	0.0086	2.8376	IL1R1, MYD88, NTSR2, SIRPB1, VIPR1, TNFRSF10B, IFNAR2, CCL2, EDN3, AGT

KEGG Pathways (Top 5)					
Term	Count	%	*p*-value	Fold Enrichment	Genes
GO:0045444∼fat cell differentiation	7	1.632	0.0090	3.8699	NR4A2, FOXO1, GDF10, METRNL, NR4A1, C1QTNF3, CEBPD
hsa04060:Cytokine-cytokine receptor interaction	14	3.26	2.97E-05	4.0800	GDF10, NGFR, IL1R1, TNFSF4, CXCL14, CSF1, TNFSF10, IL33, TNFRSF10B, IFNAR2, GDF9, CCL2, TNFRSF1A, IL17RB
hsa04080:Neuroactive ligand-receptor interaction	17	3.96	3.93E-05	3.3373	VIPR1, LTB4R, AGT, P2RX5, CHRNB4, NTSR2, GLRA2, GABRD, GPR83, GRID1, SSTR2, PRSS3, PARD3, GRM5, EDN3, LPAR4, GABRA6
hsa04010:MAPK signaling pathway	21	4.9	4.30E-04	2.3841	ERBB3, FGFR3, NGFR, PDGFRB, CACNA2D1, EFNA1, GADD45B, JUN, CACNG4, FOS, FGF5, RPS6KA1, IL1R1, MYD88, CSF1, HSPA1A, HSPA1B, FLT1, PDGFC, NR4A1, TNFRSF1A
hsa04950:Maturity onset diabetes of the young	4	0.93	7.94E-04	18.8458	PAX6, HES1, NKX2-2, NEUROD1
hsa04061:Viral protein interaction with cytokine and cytokine receptor	6	1.4	8.30E-04	7.7096	CXCL14, CSF1, TNFSF10, TNFRSF10B, CCL2, TNFRSF1A

### Limitations

There are a number of limitations inherent to current methods in postmortem studies. PMI has been extensively reported on as a factor in postmortem RNA degradation, however, a recent report (K. White et al., 2018) on tissue collected from the NIH Neurobiobank concludes that RIN numbers are a more reliable indicator of RNA quality than PMI and that a RIN number of six or above is acceptable for postmortem RNA research. However, our tissue was stained and ran through alcohol dehydration for the laser capture procedure, therefore, a few cases dropped to a RIN of four or lower and we chose to include cases as low as 4, although most were above 6. Our RNA-seq kit was specifically chosen for use with lower quality samples. Although samples with a RIN score close to four show some degradation, the use of short read next generation sequencing still facilitates the identification and quantification of gene expression. A recent comparative study showed robust gene expression analysis even in lower quality and low input samples using ribodepletion library preparation and short read NGS (Schuierer et al., 2017). Cause of death may also influence RNA degradation, but would be represented in both ASD cases and controls. Limitations in the sample number due to the paucity of postmortem samples in ASD coupled with the work intensive isolation of individual PCs have made it difficult to analyze differences in age, sex and cause of death. With regards to age at donation and PMI, the two groups were matched as best as possible based on the available tissues. Overall, the age and PMI between the ASD cases and controls were not significantly different.

The data suggest that PCs are neurons of interest in ASD with the number of differentially expressed genes on par with that reported in cortical cells. More cases with a method conducive to gathering larger quantities of isolated PCs would be important future studies to elucidate developmental and gender differences. Additionally, larger sample sizes will allow for multiple testing correction to identify differentially expressed genes. Here, we used a *p*-value of <0.05 as a cutoff, which clinical trials have suggested as an appropriate cutoff for pilot studies (Lee et al., 2014) and focused on the pathway enrichment, which inputs nominally significant results. When using a false discovery rate with a threshold of 0.2, four genes were differentially expressed: CCL2, ZFP36, ADAMTS1 and GALNT17. Future studies should compare transcriptional profiles to the genetic mutations implicated in each sample and it would be useful to compare the PC transcriptional profile to other cell types in ASD tissue, such as granule cells, to determine whether PCs are more heavily impacted. Substantial literature exists relating to PCS and the potential function of many of the implicated genes, particularly from rodent studies, however, a full discussion is outside the scope of this study. Additionally, validation of gene targets of interest with immunohistochemistry, *in situ* hybridization or qRT-PCR would be valuable. Genes implicated in ASD are highly heterogeneous, often having significant statistical association but unclear functional impact. Therefore, we chose to base our analyses and discussion on shared pathways through which these genes may participate in the etiology of ASD.

## Discussion

### Connectivity/nervous system development

Social interaction, language and coordinated sensorimotor behaviors depend on the integration of information across brain areas. In ASD, hyper- and hypo-connectivity between regions have been extensively reported in neuroimaging studies (Belmonte et al., 2004; Just et al., 2004; Just et al., 2007; Minshew and Williams, 2007; Mostofsky and Ewen, 2011; Wass, 2011; Mak-Fan et al., 2013; Nair et al., 2014; Ray et al., 2014; Dean et al., 2016; Jahedi et al., 2017). The cerebellum in particular has been reported to have volumetric differences based on connectivity trajectories during development (Becker and Stoodley, 2013; Hampson and Blatt, 2015; Wang et al., 2014; Scott et al., 2009; Arnold Anteraper et al., 2019). These findings suggest the possibility of a disrupted developmental trajectory of white matter development in ASD.

The transcriptome findings presented here suggest that PCs specifically may be contributing to altered connectivity in ASD. This implication is consistent with large-scale GWAS and gene pathway enrichment findings implicating alterations in neurite outgrowth and guidance in ASD (Hussman et al., 2011). PCs are the only output neurons of the cerebellar cortex, sending their axons to the deep cerebellar nuclei to coordinate information to the rest of the brain. Downregulation of genes in pathways such as cell migration, axon guidance, nervous system development and neuron projection/extension cell proliferation pathways could have significant consequences for the functional organization of the cerebellum. During development, cell-adhesion molecules and extracellular matrix organization are critical for patterning the intricate zones and stripes of the cerebellar cortex (Sotelo, 2020; Apps et al., 2018; Sillitoe and Joyner 2007; White and Sillitoe, 2013). How these downregulated genes impact the early development of PC patterning and their potential long-term impact on functional connectivity in the adult brain will be important to determine. In an analysis of PC gene expression in the developing mouse brain (Clifford et al., 2019), identified a cluster of genes enriched for genes associated with ASD whose expression was positively regulated during PC development. Gene ontology analysis showed this cluster of genes was enriched for those involved in cell-cell signaling, synaptic transmission, cerebellar PC layer development, cellular localization and developmental maturation. Although this analysis was performed in mice at varying developmental stages, we identify gene enrichment in similar pathways, supporting the validity of our findings in human PCs.

### Extracellular matrix organization

A consistent finding in genome-wide association studies (GWAS) is genetic susceptibility in ECM-associated genes (Weiss and Arking, 2009; Hussman et al., 2011; Wang et al., 2009; Anney et al., 2010). Among these genes, Reelin, a disintegrin and metalloproteinase with thrombospondin motifs (ADAMTS) and semaphorins provide the strongest evidence linking ASD to disruption of the ECM (for review see: Pantazopoulos and Berretta, 2016; Sorg et al., 2016). Considering that these GWAS studies typically analyze DNA from whole blood, it is encouraging that mRNA expression data from a specific neuron subtype can recapitulate these findings and show differential mRNA expression of previously implicated genes. Notably, RELN, as well as multiple ADAMTS and semaphorin genes were differentially expressed in ASD compared to neurotypical individuals in our PC transcriptome analysis.

Components of a condensed form of the ECM, perineuronal nets (PNNs), are highly enriched in the deep cerebellar nuclei (DCN), surrounding synapses from PCs onto DCN neurons and have recently been shown to be important for cerebellar plasticity and the formation of associative memories (Carulli et al., 2020). PCs produce various components of PNNs and are themselves surrounded by a loose (semi-organized) form of PNN (Carulli et al., 2006). PNNs are typically associated with fast-spiking neurons, have numerous functional roles in development, plasticity and neuroprotection and can be modified by numerous factors including metalloproteinases (for review see: Fawcett et al., 2019). ADAMTS are a type of metalloproteinase shown to degrade PNN components and are associated with remodeling and excitotoxicity. The differential expression of several ADAMTS in ASD PCs, suggests that both PC function and input to the DCN may be impacted.

PNNs have gained attention due to their dysregulation in multiple psychiatric conditions (Berretta et al., 2015; Sorg et al., 2016; Wen et al., 2018) and they have known roles in modulating parvalbumin interneuron function and gamma oscillations. As there is increasing evidence of association between parvalbumin interneuron dysfunction and ASD, the study of PNNs in the context of ASD will be important for future research (Burket et al., 2017; Brandenburg and Blatt 2022).

### PC function and synaptic signaling

Parvalbumin, a calcium-binding protein, has been reported to be dysregulated in multiple mouse models of ASD (Gogolla et al., 2009; Wöhr et al., 2015) as well as in human postmortem studies (Hashemi et al., 2017; Brandenburg and Blatt 2022). Notably, while PC loss is commonly observed in ASD, the remaining PCs in postmortem ASD cases have shown reduced mRNA expression for parvalbumin using *in situ* hybridization histochemistry (Soghomonian et al., 2017). This finding suggests brain-wide changes in inhibitory network function. However, PCs have an exceptionally high concentration of several different calcium-binding proteins that are critical for PC calcium homeostasis (Schwaller et al., 2002). Although our cases did not show significant differences in parvalbumin expression, calcium ion response was an enriched pathway for downregulated genes.

A potential relationship exists between calcium ion response and the ECM pathways. The physiochemical properties of ECM/PNNs can regulate concentration gradients by acting as a reservoir for extracellular ions and modifying their diffusion properties (Morawski et al., 2015). If ECM/PNNs are indeed dysregulated around PCs in ASD, they could potentially impact ion homeostasis and produce differences in calcium ion response and neuronal signaling. As calcium homeostasis is an important aspect of PC function, the possibility of ECM/PNN disruption may be among important pathways to consider in ASD.

Several pathways related to functional properties of PCs were enriched, including signal transduction, regulation of membrane potential, positive regulation of phosphatidylinositol 3-kinase (PI3K) signaling and the mitogen-activated protein kinase (MAPK) signaling pathway. The interplay of these various pathways and the functional outcomes of their disruption may provide clues for treatment strategies, especially in the context of sensorimotor challenges. The MAPK signaling pathway was identified in the KEGG pathway enrichment analysis. Alterations in MAPK signaling have been associated with altered brain cytoarchitecture (Pucilowska et al., 2015; Pucilowska et al., 2018) and can result in social deficits and cognitive dysfunction reminiscent of ASD (Faridar et al., 2014; Yufune et al., 2015). Likewise, PI3K signaling plays an important role in neuronal guidance and circuit formation (Hussman et al., 2011). Among the genes in this pathway, loss of PDGFRB expression has been associated with social interaction deficits (a core feature of ASD) in mouse models (Nguyen et al., 2011). Additionally, loss of CACNA2D1 (another gene differentially expressed between the ASD and control PCs) expression altered the morphology and functionality of cerebellar PCs (Barclay et al., 2001; Brodbeck et al., 2002). (Velmeshev et al., 2019) performed single nuclei RNAseq from individuals with ASD (15 individuals) and control (16 individuals) and showed an enrichment in differential gene expression associated with chemical synaptic transmission, axon guidance and neuronal migration. Although they performed their analysis on cells from the prefrontal cortex and anterior cingulate cortex, they identified similar GO categories as we identified here.

### Immune function

As reviewed in (Meltzer and Van De Water, 2017), immune pathways are commonly altered in ASD GWAS and transcriptome studies. However, the transcriptome studies reported typically use bulk tissue so have representation of neurons, microglia and astrocytes. In the present study, we isolated PCs from the surrounding tissue and found down-regulated genes in a variety of immune related pathways. Among these genes, CCL2 has been previously suggested to act as a modulator of neuronal activity, neuroendocrine functions and neuronal inflammatory processes (Banisadr et al., 2005). It has also been suggested to support the maturation of PCs (Meng et al., 1999)by promoting dendrite outgrowth and synapse maturation, GO processes that were shown to be enriched in the STRINGdb analysis. Experiments in mice have shown that IRF7 (interferon regulatory factor 7) was involved in toll like receptor-mediated neuronal protection from stressors, such as ischemia (Stevens et al., 2011). Additionally, the deletion of IRF-1 in mice lead to elevated levels of cognitive impairment, supporting a role for IRF1 in cognitive function (Mogi et al., 2018). Mouse knock-out studies have shown that Interleukin one receptor 1 (IL1R1) has also been associated with social interactivity and working memory/cognition in mice (DiSabato et al., 2021). Myeloid differentiation primary response protein 88 (MyD88) knockout in mice resulted in decreased motor activity and elevated levels of anxiety-like behavior. In addition, the MyD88^−/−^ mice showed decreased neuronal arborization (Schroeder et al., 2021). Myd88 −/− neurons were unable to respond to Toll-like receptor 3 (TLR3) activation leading to an inhibition of dendritic growth (Chen et al., 2017). These results and others show that immune genes play key roles in neuronal development and maturation leading to phenotypes that are consistent with ASD.

Immune related genes may also have a potential relationship with ECM pathway dysregulation, as matrix metalloproteinases (MMPs) can induce neuroimmune responses by triggering microglial activation and cytokine release (Bitanihirwe et al., 2016). It is possible that dysregulation of ECM/PNNs, consistent with the pathway findings reported here, could interact with immune pathways in ASD, with a combination of environmental factors and genetic susceptibility contributing to an altered transcriptional profile of ECM/PNN genes.

### Summary

Our discussion centers on the top enriched GO pathways and potential evidence for their interaction with other implicated pathways, but a definitive link to any particular pathway is speculative in ASD cases. We hope future studies will validate individual genes from this pilot study and examine their participation in the broad pathways hypothesized. It is worth noting that specific sets of genes are often dysregulated in ASD (Cell, 2013, 155 (5), 1,008–1,021;, Cell, 2013, 155 (5), 997–1,007; Velmeshev et al., Science (New York, N.Y.), 2019, 364 (6,441), 685–89), suggesting that gene regulatory mechanisms are impacted. Chromatin modifiers are capable of regulating ASD risk genes (Murgatroyd and Spengler, 2012; Krumm et al., 2014; Hoffmann and Spengler, 2021) and their contribution to the differential expression of genes in PCs will be important to uncover. Although the individuals in this cohort were adolescents or adults at the time of death, it is noteworthy that many of the enriched pathways suggest altered PC developmental trajectories and connectivity in ASD cases. The impact of early developmental transcript differences on PC specification within the complex landscape of cerebellar microcircuitry maturation may therefore be a worthwhile avenue of exploration. Other enriched pathways that impact mature PCs, such as signal transduction, regulation of membrane potential, cellular response to calcium ions and positive regulation of phosphatidylinositol three kinase signaling may be amenable to treatment strategies. Identification of altered gene expression in PCs is a step toward understanding the complex molecular and physiological mechanisms underlying ASD.

## Data Availability

The data presented in the study are deposited in the GEO repository, accession number GEO GSE211154, https://www.ncbi.nlm.nih.gov/geo/query/acc.cgi?acc=GSE211154
