# Supplementary material for: Transcriptomic analysis of isolated and pooled human postmortem cerebellar Purkinje cells in autism spectrum disorders
Source: Front Genet. 2022 Nov 9;13:944837. doi: 10.3389/fgene.2022.944837 (PMC9683032; doi:10.3389/fgene.2022.944837)

Supplemental Figures

Transcriptomic analysis of isolated human postmortem cerebellar Purkinje cells in autism spectrum disorders

Cheryl Brandenburg[1,2,*], Anthony J. Griswold[3], Derek Van Booven[3], Michaela B.C. Kilander[1], Jeannine A. Frei[1], Michael W. Nestor[1], Derek M. Dykxhoorn[3], Margaret A. Pericak-Vance[3], Gene J. Blatt

Supplemental Figure 1. Principal component analysis (PCA) of the variance stabilized transformed values shows a clear separation of sex based on gene expression, but no outlier samples beyond 2.5 standard deviations from the mean nor distinction of samples by status or self-reported ethnicity.

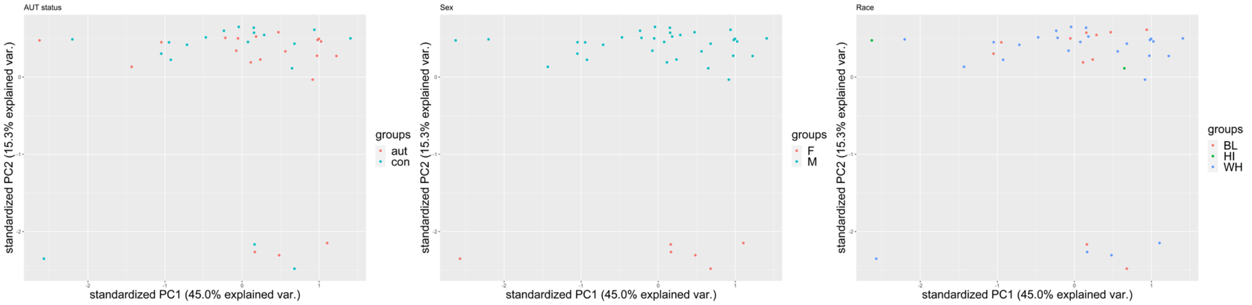

Supplemental Figure 2. Average normalized expression of genes associated with cerebellar PCs versus those associated with cerebellar astrocytes or oligodendrocytes (Kuhn et al. 2012). The average expression of the PC associated genes was significantly higher than those associated with astrocytes or oligodendrocytes (Kruskal-Wallis p-value < 0.0001).

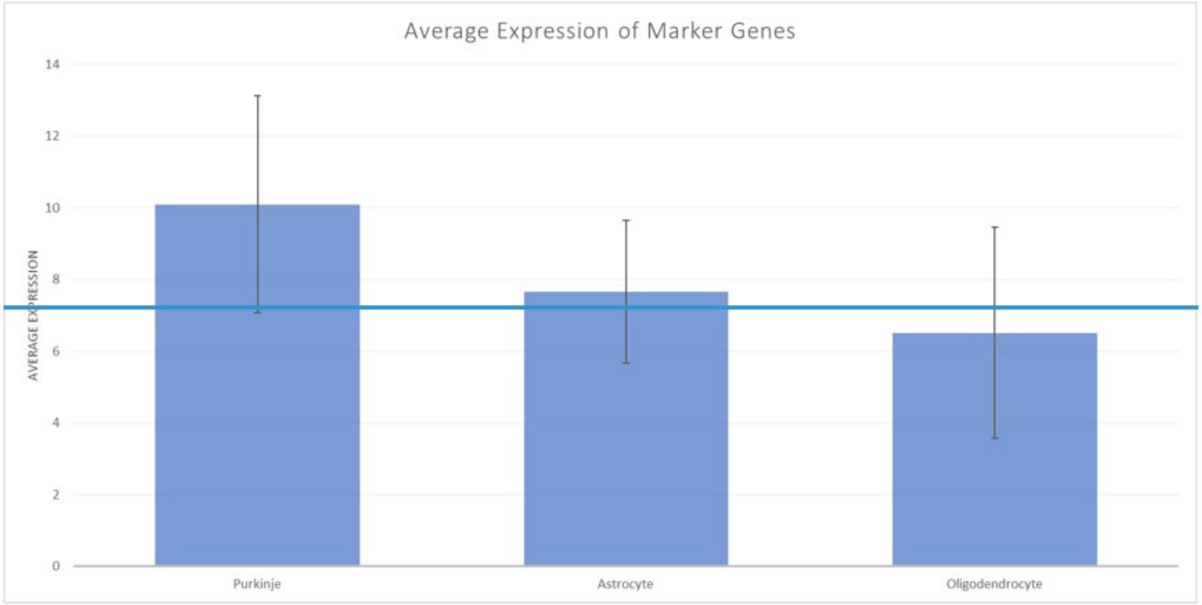

Supplemental Figure 3. Individual analysis of each of the top ten up- and down- regulated genes, showing group differences between ASD and control cases.

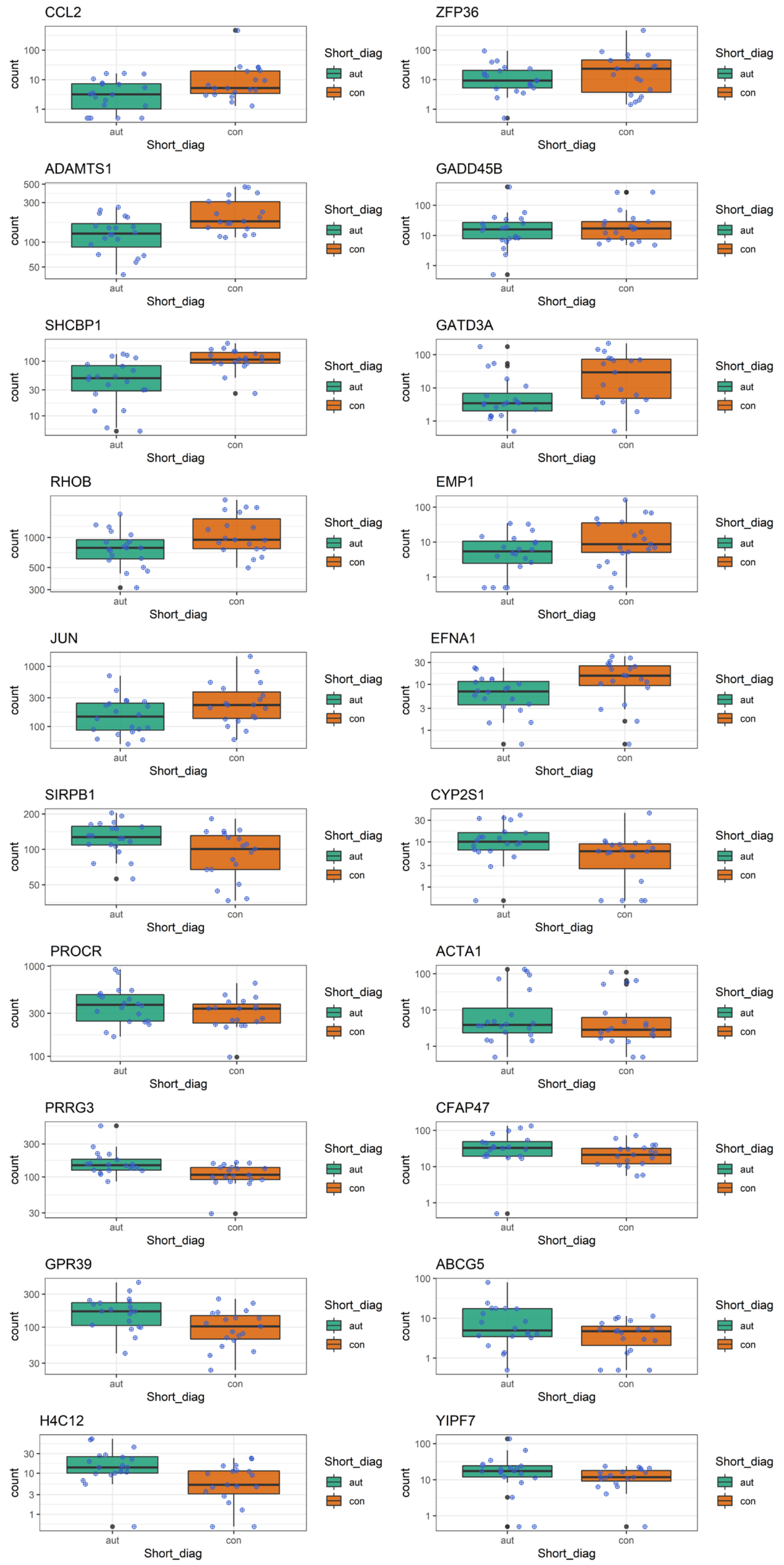

Supplemental Figure 4. Top 25 gene ontology processes.

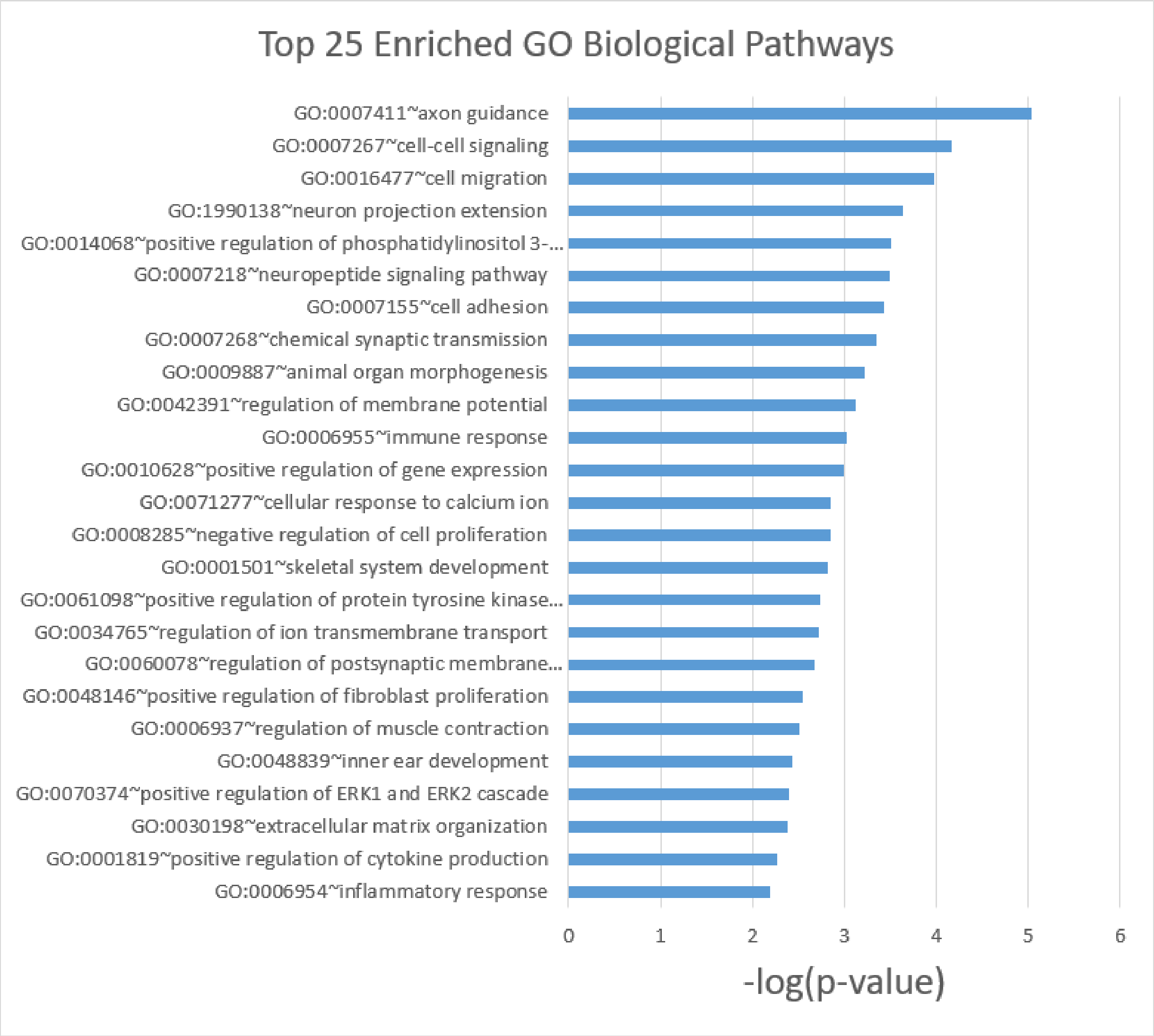

Supplemental Figure 5. Overall protein-protein interaction clusters defined by STRNG among the genes which showed decreased expression between cases and controls. Overall the interaction network was composed of 26 subclusters that had a PPI enrichment p-value < 0.005.

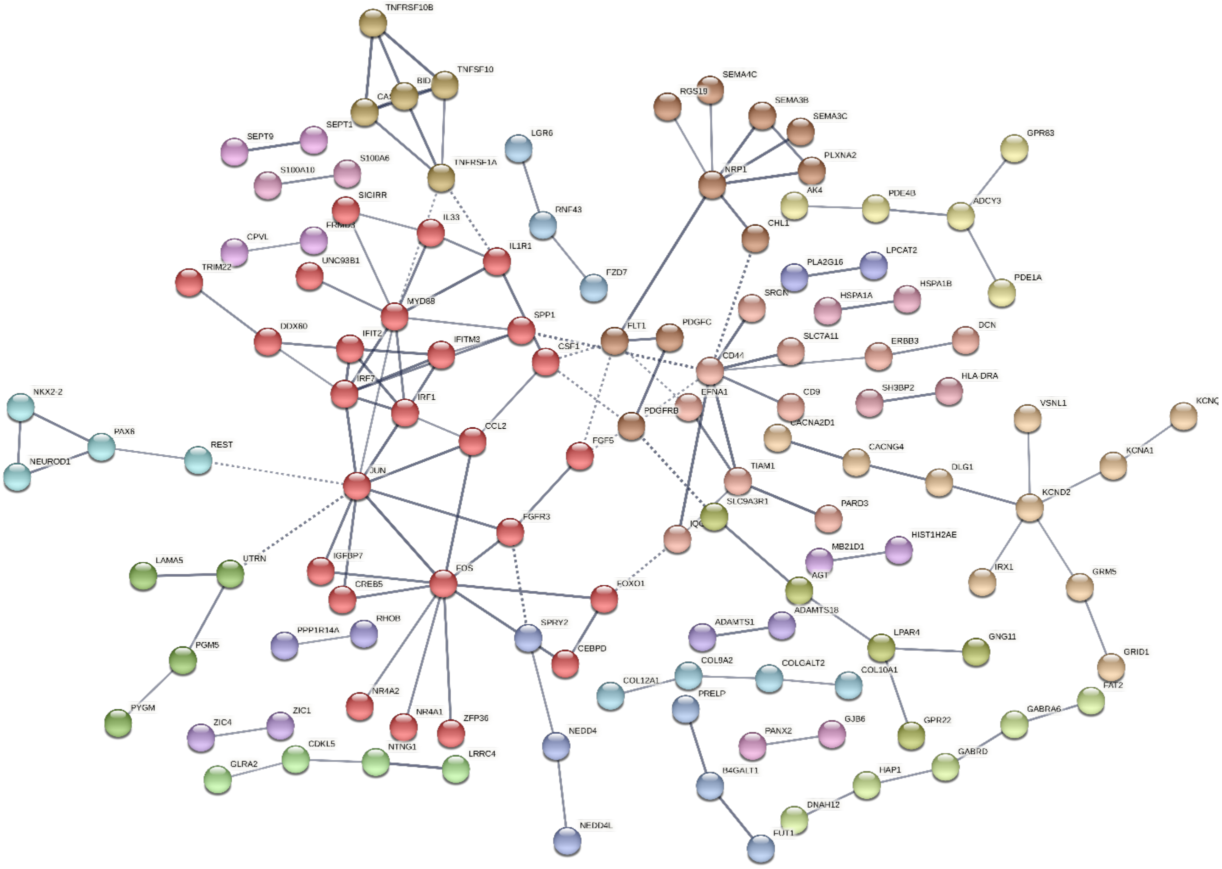

Supplement: Supplementary file 3 [file Image1.pdf]
